# PHYSICAL AND MENTAL HEALTH STATUS AMONG SANDWICH AND NONSANDWICH CAREGIVERS BY RACE/ETHNICITY

**DOI:** 10.1093/geroni/igad104.0417

**Published:** 2023-12-21

**Authors:** Christina Miyawaki, Erin Bouldin

**Affiliations:** University of Houston, Houston, Texas, United States; University of Utah, Salt Lake City, Utah, United States

## Abstract

Sandwich caregivers are middle-aged adults who care for both a child and an older adult. Using data from the Behavioral Risk Factor Surveillance System Caregiver Module in 50 jurisdictions from 2016-2021, we compared the prevalence of sandwich caregivers and their physical and mental health across racial/ethnic groups. We included 23,853 caregivers aged 45-64 years. Sandwich caregivers (N=2,486) were those who lived with a child (≤18 years) and provided care/assistance to a parent/grandparent with a long-term illness/disability during the past 30 days. The prevalence of sandwich caregiving varied by race/ethnicity, with 18.4% of non-Hispanic Asian, 14.5% of non-Hispanic Black, 11.5% of Hispanic, 10.2% of non-Hispanic White, and 9.5% of non-Hispanic other/multiracial respondents classified as sandwich caregivers. Prevalence ratios (PR) from log-binomial regression models adjusted for age, sex, hours of caregiving, and type of care were used to compare weighted estimates. Sandwich caregivers generally had similar physical health and better mental health compared to non-sandwich caregivers. Compared to non-sandwich caregivers from the same racial/ethnic background, Black sandwich caregivers had a significantly higher prevalence of excellent/very good/good health (PR=1.15) and lower frequent mental distress (PR=0.90), while Asian (PR=0.78) and White (PR=0.91) sandwich caregivers had a significantly lower prevalence of frequent mental distress. Some racial/ethnic groups of sandwich caregivers were in better physical and mental health than non-sandwich caregivers. This could reflect a need for a higher level of physical and mental health to take on a sandwich caregiving role. Caregiver supports should consider sandwich caregivers’ unique characteristics and their racial/ethnic backgrounds.

